# Supplementary material for: Development of a Hallmark Pathway-Related Gene Signature Associated with Immune Response for Lower Grade Gliomas
Source: Int J Mol Sci. 2022 Oct 9;23(19):11971. doi: 10.3390/ijms231911971 (PMC9570050; doi:10.3390/ijms231911971)
Supplement: Supplementary file 1 [file ijms-23-11971-s001.zip › ijms-1943710-supplementary.pdf]

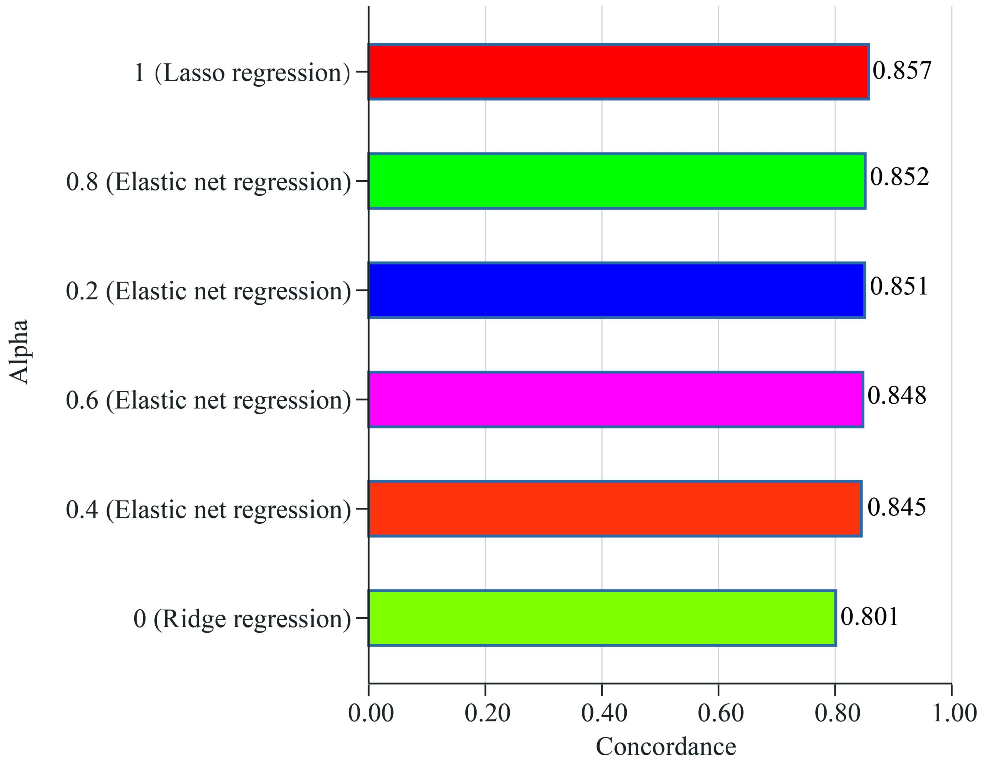


**Figure S1.** The comparison of LASSO, Ridge and Elastic net regressions in the prediction of prognosis for LGGs.

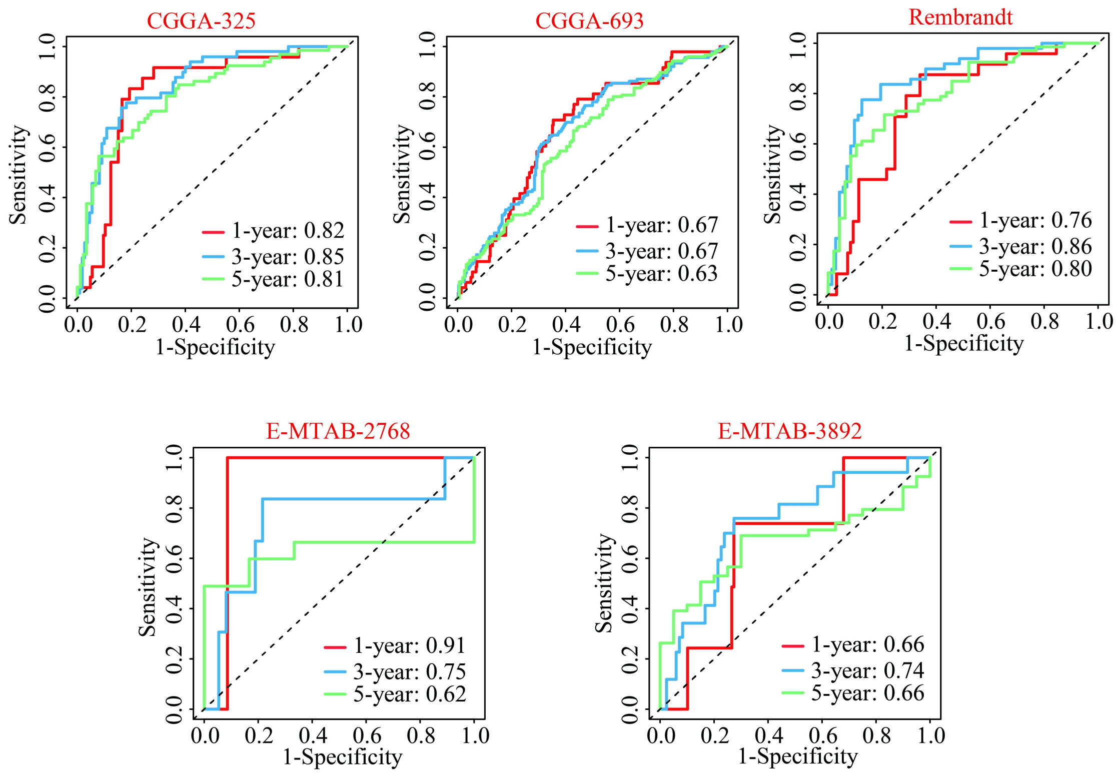


**Figure S2.** The AUC of 1-, 3-, and 5-year OS in CGGA-325, CGGA-693, Rembrandt,

E-MATB-2768 and E-MATB-3892 datasets, respectively.

# ABI1

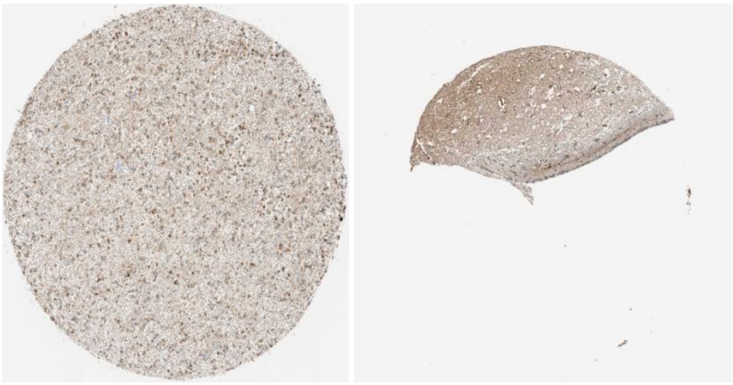

**CAB008375**

LGG tissue

Staining: Medium

Intensity: Moderate

Quality: > 75%

**CAB008375**

Normal tissue

Staining: Low

Intensity: Moderate

Quality: < 25%

# BRCA1

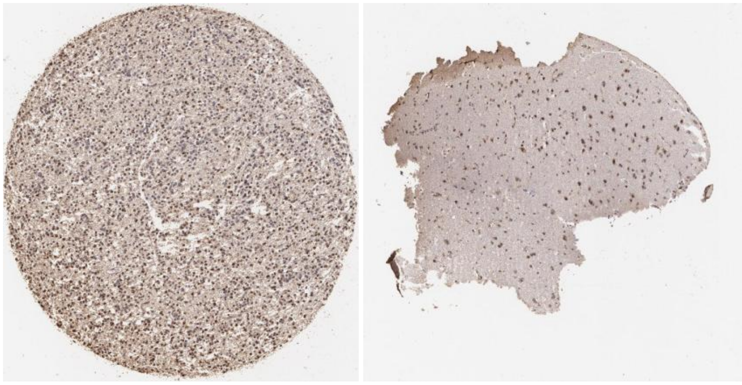

**CAB001946**

LGG tissue

Staining: Medium

Intensity: Moderate

Quality: 75% ~ 25%

**CAB001946**

Normal tissue

Staining: Low

Intensity: Moderate

Quality: < 25%

# CAPG

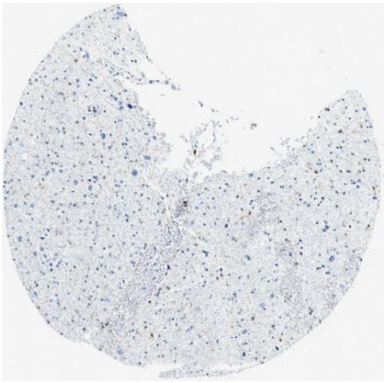

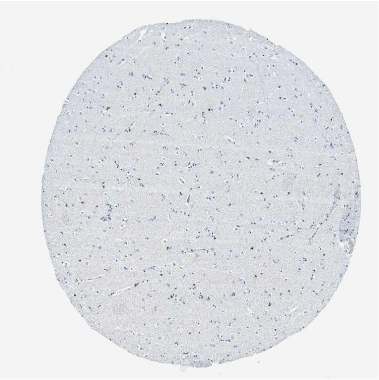

**HPA019092**

LGG tissue

Staining: Low

Intensity: Moderate

Quality: < 25%

**HPA019092**

Normal tissue

Staining: Not detected

Intensity: Week

Quality: < 25%

# EIF3D

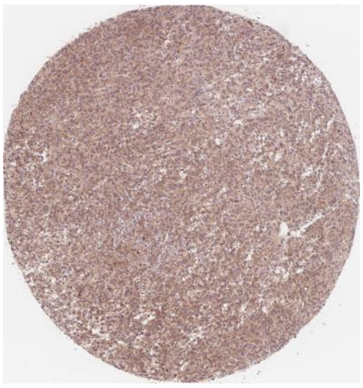

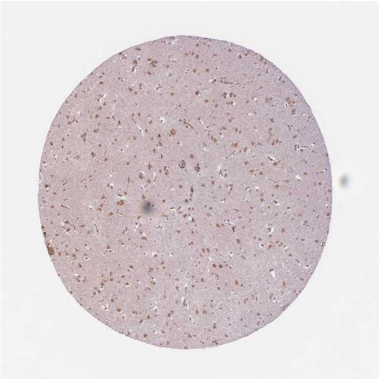

**HPA066216**

LGG tissue

Staining: Medium

Intensity: Moderate

Quality: > 75%

**HPA066216**

Normal tissue

Staining: Low

Intensity: Moderate

Quality: < 25%

# FLNA

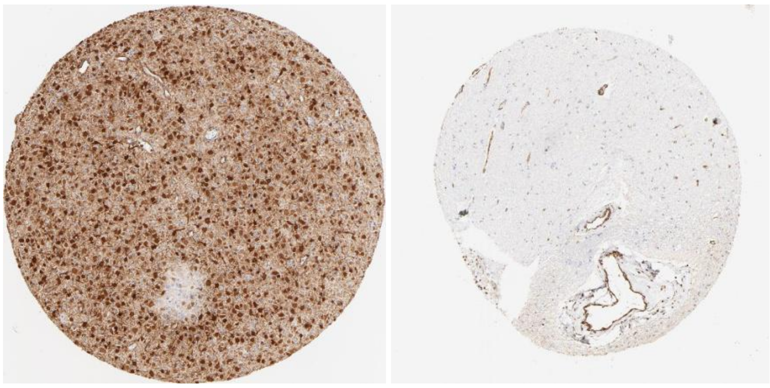

| **HPA002925** | **HPA002925** |
| --- | --- |
| LGG tissue | Normal tissue |
| Staining: High | Staining: Low |
| Intensity: Strong | Intensity: Weak |
| Quality: > 75% | Quality: > 75% |

**Figure S3.** The expression of five AR-related genes between LGG and corresponding normal tissues at the protein level.

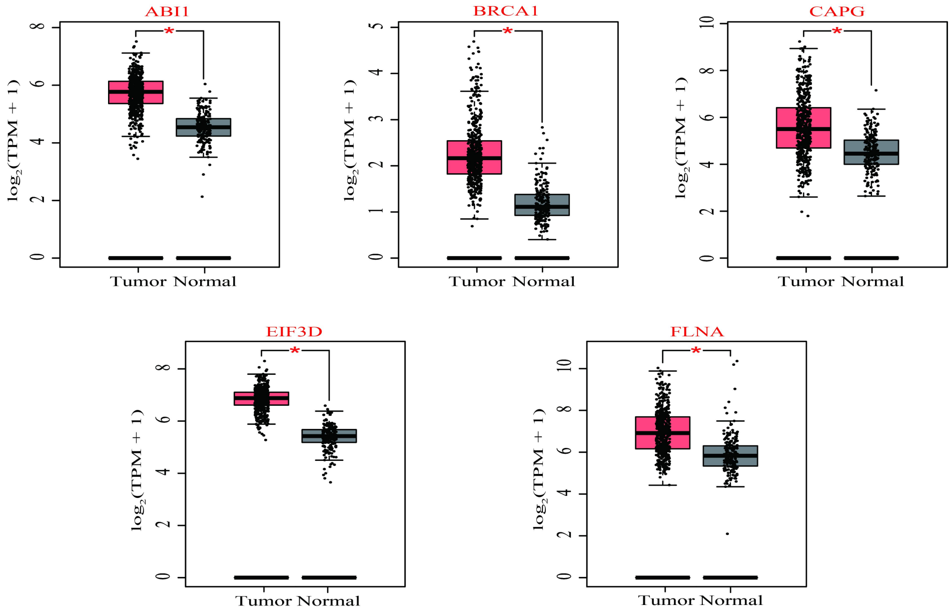


**Figure S4.** The expression of five AR-related genes between tumor and normal tissues via GEPIA database.

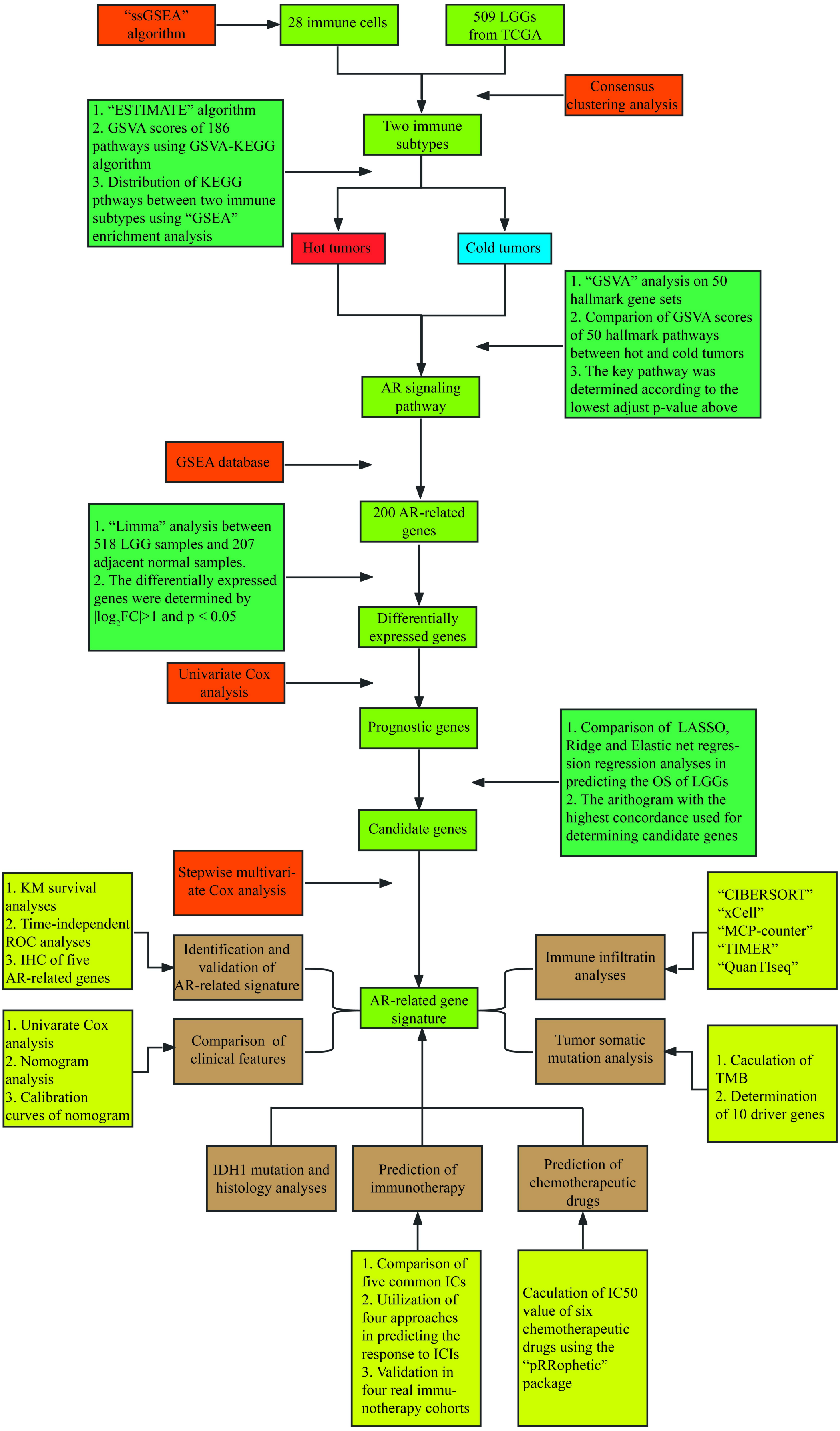


**Figure S5.** The flow chart in this study.
